# Accuracy Evaluation of Collapsed Cone Convolution Superposition Algorithms for the Nasopharynx Interface in the Early Stage of Nasopharyngeal Carcinoma

**DOI:** 10.1155/2022/5227609

**Published:** 2022-05-28

**Authors:** Yuan-Chun Lai, Li-Chung Hung, Chien-Chung Jeng, Chen-Ju Feng, Tung-Hao Chang, Shih-Ming Hsu

**Affiliations:** ^1^Department of Radiation Oncology, Changhua Christian Hospital, Changhua, Taiwan; ^2^Department of Medical Imaging and Radiological Sciences, Central Taiwan University of Science and Technology, Taichung, Taiwan; ^3^Department of Physics, National Chung Hsing University, Taichung, Taiwan; ^4^Department of Biomedical Imaging and Radiological Sciences, National Yang Ming Chiao Tung University, Taipei, Taiwan; ^5^Department of Medical Imaging and Radiological Technology, Yuanpei University of Medical Technology, Hsinchu, Taiwan

## Abstract

This study combined the use of radiation dosimeteric measurements and a custom-made anthropomorphic phantom in order to evaluate the accuracy of therapeutic dose calculations at the nasopharyngeal air-tissue interface. The doses at the nasopharyngeal air-tissue interface obtained utilizing the Pinnacle and TomoTherapy TPS, which are based on collapsed cone convolution superposition (CCCS) algorithms, were evaluated and measured under single 10 × 10 cm^2^, 2 × 2 cm^2^, two parallel opposed 2 × 2 cm^2^ and clinical fields for early stage of nasopharyngeal carcinoma by using EBT3, GR-200F, and TLD 100. At the air-tissue interface under a 10 × 10 cm^2^ field, the TPS dose calculation values were in good agreement with the dosimeter measurement with all differences within 3.5%. When measured the single field 2 × 2 cm^2^, the differences between the average dose were measured at the distal interface for EBT3, GR-200F, and TLD-100 and the calculation values were -15.8%, -16.4%, and -4.9%, respectively. When using the clinical techniques such as IMRT, VMAT, and tomotherapy, the measurement results at the interface for all three techniques did not imply under dose. Small-field sizes will lead to dose overestimation at the nasopharyngeal air-tissue interface due to electronic disequilibrium when using CCCS algorithms. However, under clinical applications of multiangle irradiation, the dose errors caused by this effect were not significant.

## 1. Introduction

Radiotherapy is the main treatment method for nasopharyngeal carcinoma (NPC). However, the presence of several important organs adjacent to the nasopharynx, such as the optic chiasma, brainstem, spinal cord, and parotid gland, increases the difficulty of radiotherapy. Conventional external beam radiotherapy often leads to side effects and has an adverse impact on the patient's quality of life. In recent years, several new treatment techniques have been developed, including intensity-modulated radiotherapy (IMRT), volumetric-modulated arc therapy (VMAT), and tomotherapy. These techniques mainly involve stacking a large number of small fields to form a large field with different dose intensities, which allows for a more conformal dose curve surrounding the tumor, thereby lowering the dose to normal tissues and organs [1, 2]. This greatly reduces the probability of side effects in NPC patients.

Many studies have shown that tissue inhomogeneities, due to air cavities, lead to dose reduction at the air-tissue interface [3–8]. Because the scope of NPC treatment involves the nasopharyngeal cavity, this would be expected to affect the accuracy of dose calculations in the treatment planning system (TPS), with the magnitude of the dose reduction dependent on the cavity volume, field size, and photon energy. This effect is greater with a smaller field size, higher energy, and larger cavity volume [3, 5–7]. This is primarily due to the electronic disequilibrium effect at the air-tissue interface in the presence of air cavities, leading to a dose reduction at the air-tissue interface. For NPC patients, such interface regions may contain tumor cells, which could increase the probability of future tumor recurrence. Therefore, understanding the effect of the nasopharyngeal cavity on the accuracy dose calculations in a clinical TPS is necessary.

Monte Carlo simulation is one of the reliable methods to evaluate the dose of build-up area and heterogeneous substance. In order to simulate the effect of simple irradiation conditions (single field or rectangular field) on dose prescription, most of the studies have generally involved the measurement of simple fields or an applied Monte Carlo simulation. Zhao et al. further evaluated the accuracy of clinical tomotherapy treatment plans in the heterogeneous phantom against Monte Carlo simulation and measurement [9]. Haga et al. utilized XVMC algorithm that is a fast Monte Carlo calculation algorithm to compare with the dose of clinical VMAT treatment plans in the homogeneous and heterogeneous phantom [10]. Their works revealed that the commercial Pinnacle and TomoTherapy TPS provide acceptable dose accuracy in most condition and the Monte Carlo simulation is a trustworthy and feasible method to verify clinical dose, especially for inhomogeneous and low-dose region.

Our main concern was regarding the effect of the nasopharyngeal cavity on the actual dose delivery during the treatment of early stage NPC in a clinical setting. The nasopharyngeal cavity in this stage would not like the advanced cases to be filled with tumor tissue, and the maximum cavity volume could be maintained. Hence, in this study, ultrathin dosimeters, such as GR-200F, TLD-100, and EBT3 radiochromic film, were used to conduct dosimetric measurements in order to evaluate the accuracy of dose calculations for the nasopharyngeal cavity during clinical treatment, using the commercial Pinnacle and TomoTherapy TPS, which utilize collapsed cone convolution superposition (CCCS) algorithms. The aim of this study was to understand the effect of the nasopharynx on TPS dose calculations.

## 2. Methods and Materials

### 2.1. Phantom Design

**Figure 1:**
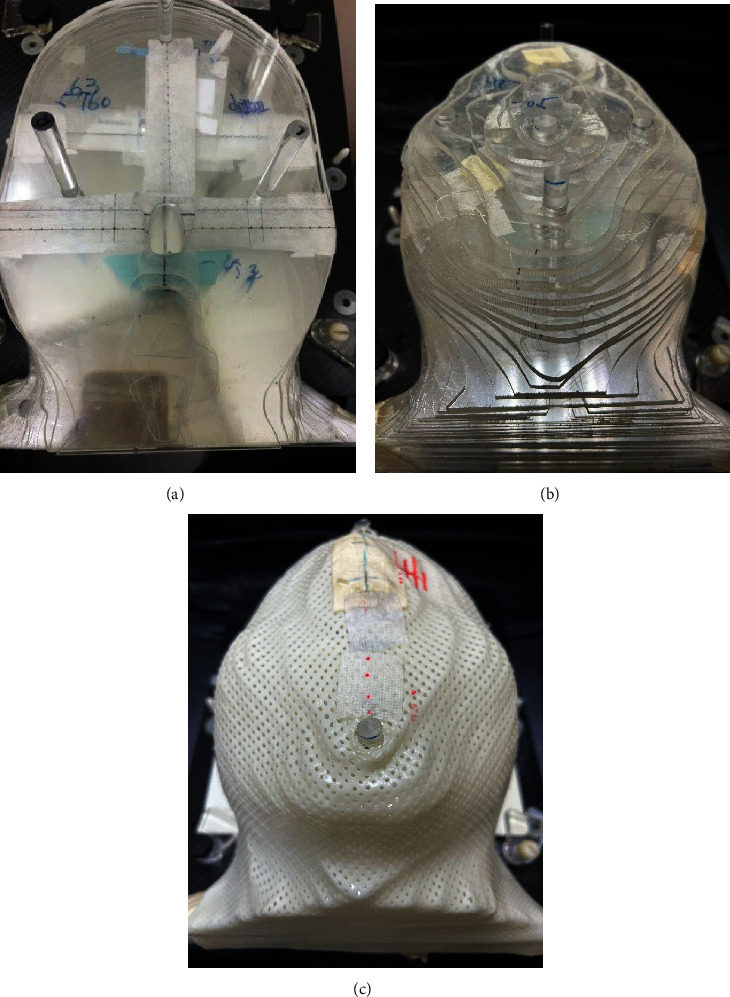
Custom-made humanoid head acrylic phantom. (a) Phantom coronal section; (b) head phantom composed of all coronal sections; and (c) head phantom fixed with a mask.

A custom-made anthropomorphic acrylic phantom of the head was created by milling machine according to coronal computed-tomography (CT) images of patients. The phantom contained an air cavity similar in shape to the nasopharyngeal cavity of the patient. This custom-made anthropomorphic phantom consisted of 47 coronal sections in slices, which were fixed in place using five acrylic cylinders. EBT3 radiochromic film and thermoluminescent dosimeters (TLD) could be placed on these coronal slices in order to measure the radiation dose and dose distribution. The slice thickness at the tumor site was 5 mm, as shown in Figure 1.

### 2.2. Treatment Plan

**Figure 2:**
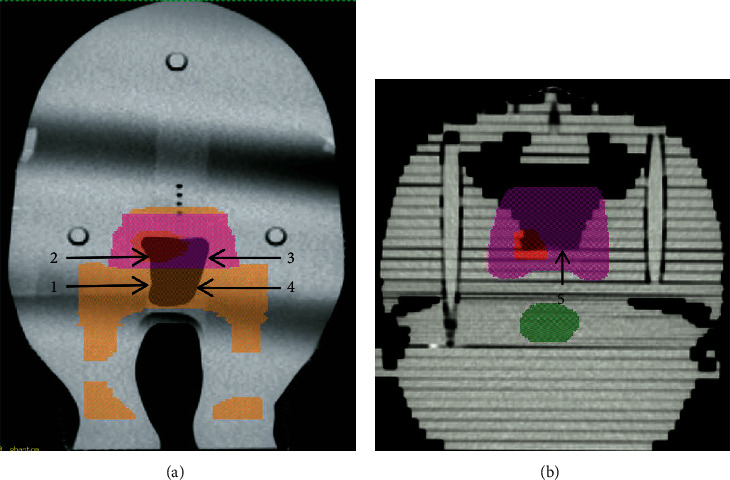
TLD placement points: (a) coronal view and (b) axial view. Red represents the GTV, pink represents the PTV1, orange represents the PTV2, and dark green represents the brainstem.

CT (LightSpeed GE, USA) was used to acquire images of the phantom using a CT slice thickness of 1.25 mm. The images were transferred to Pinnacle TPS (Pinnacle3® version 9.8 Philips, USA) and TomoTherapy TPS (TomoTherapy Planning Station Hi-ART ® version 4.3.2 Accuray, USA). Pinnacle TPS and Tomotherapy TPS based on CCCS are the commonly used commercial treatment planning system for linear accelerator and tomotherapy to treat NPC disease, respectively. A clinical radiation oncologist defined the position and treatment range of the NPC with tumor stage T1N0M0, including the gross target volume (GTV), clinical target volume 1 (CTV1), and clinical target volume 2 (CTV2). A 3-mm margin was used to form the planning target volume (PTV). The positions of the normal tissues and organs were also specified (Figure 2). The GTV, PTV1, and PTV2 were prescribed doses of 7200 cGy, 6480 cGy, and 5940 cGy, respectively. Pinnacle TPS was used to create the treatment plan required to implement VMAT and IMRT. The Elekta Axesse (Sweden) linear accelerator was employed, together with the Agility 5 mm leaf width multileaf collimator (MLC), using 6 MV photon beams. TomoTherapy TPS was used to calculate the treatment plan required for tomotherapy using 6 MV photon beams.

In order to evaluate the effect of actual nasopharyngeal cavity on the simple irradiation conditions, the beam with 10 × 10 cm^2^ and 2 × 2 cm^2^ single field was applied from the direction of 90° individually to assess the dose accuracy of the proximal and distal interface. Beams with 2 × 2 cm^2^ opposing parallel were irradiated from directions of 90° and 270°, respectively, to assess the dose accuracy of interfaces. The inverse of the build-up ratio (BUR^−1^) is defined as the ratio of the dose at the interface to the dose at the depth of maximum dose in rebuild-up region which is also used to evaluate the central axis dose distribution of 2 × 2 cm^2^ single field [11].

The nine beam angles applied in IMRT were 165°, 130°, 80°, 40°, 0°, 320°, 280°, 230°, and 195°. The minimum segment area was set at 4 cm^2^, the minimum segment monitor unit (MU) was set at 3, and the dose rate was 600 MU/min. The beams applied in VMAT were counterclockwise from 176° to 184° with a collimator angle of 350°, and clockwise from 184° to 176° with a collimator angle of 10°. The maximum dose rate was 600 MU/min and the dose grid of the three axes set in the TPS was 3 mm. Furthermore, 6 MV photon beams were used for tomotherapy, and the parameters were as follows: modulation factor, 3.5; field width, 2.5 cm; pitch, 0.215; maximum dose rate, 900 MU/min; and the fine dose grid setting in the TPS.

### 2.3. Dose Algorithm

Pinnacle and TomoTherapy TPS both employ CCCS algorithms for dose calculations. This algorithm is able to correct for the influence of tissue inhomogeneity, patient surface contours, and beam modifiers based on correcting the energy fluence. The system calculates the total energy released per unit mass (TERMA) of each monoenergetic beam after entering the tissue. The TERMA is then convolved with the dose kernel to calculate the dose deposit caused by the beams. The dose kernel spread function of various monoenergetic beams that the TPS used to calculate dose is generated in water by Monte Carlo method with the assumption of the existence of electronic equilibrium. Finally, total incident energy in the energy spectrum is summarized to obtain the dose [12–14]. The following equation describes the dose distribution:

(1) $$\begin{array}{c} \text{D}\left(\overset{⟶}{\text{r}}\right)=\int_{\text{V}}\frac{\mu }{\rho }\left({\overset{⟶}{\text{r}}}^{\prime }\right)\Psi \left({\overset{⟶}{\text{r}}}^{\prime }\right)\text{K}\left(\overset{⟶}{\text{r}}-{\overset{⟶}{\text{r}}}^{\prime }\right){\text{d}}^{3}\overset{⟶}{\text{r}}, \end{array}$$

where $K\left(\overset{⟶}{r}-{\overset{⟶}{r}}^{\prime }\right)$ is the convolution kernel, the energy fluence at the depth r′, $\Psi \left({\overset{⟶}{r}}^{\prime }\right)$, multiplied by the energy attenuation coefficient, $\mu /\rho \left({\overset{⟶}{r}}^{\prime }\right)$, represents the TERMA at depth r′. As the beam gets into low density areas such as air cavity, the dose kernel distribution is then corrected using density scaling for heterogeneous medium. Previous research has shown that the algorithm will overestimate the dose at the interface between air and soft tissues for small-field beams, with more severe overestimation for smaller field sizes [15].

### 2.4. Radiation Dose Measurements

This study used GR-200F (LiF:Mg,Cu,P, Hangzhou Freq-Control Electronic Technology Ltd, China) and Harshaw TLD-100 (LiF:Mg,Ti, Thermo, USA) to measure the surface dose at the interface between the nasopharyngeal cavity and tissues. The measurement points are shown in Figure 2. The five-time measurements were performed at each point. GR-200F is a highly sensitive, ultrathin TLD with a diameter of 0.5 cm. The TLD powder with thickness of about 0.1 mm (effective thickness: 5 mg·cm^−2^) and density of 2.675 g·cm^−3^ was coated and adhered to one side of PE supported layer and is suitable for measuring surface doses [16]. The reader used was the Rexon UL-320 (Rexon, USA). TLD-100 has dimensions of 3.2 × 3.2 × 0.38 mm^3^ and a density of 2.64 g·cm^−3^; a Harshaw 5500 TLD was used as a reader (Thermo, USA).

EBT3 radiochromic film (Ashland, USA) was also used in this study to measure the dose distribution on the coronal plane and the dose at the interface of the tumor site. An Epson Expression 10000XL scanner was used to scan the film using a red scanning light source. FilmQA™ software (version 2.2) was then used to analyze the coronal dose, followed by gamma evaluation to determine the difference between the two-dimensional doses measured using the EBT3 film and the treatment plan. Since the purpose of this study was to investigate the dose perturbance of nasopharyngeal cavity, the range of gamma analysis was focused on the high-dose area, 8 × 8 cm^2^, including the nasopharyngeal cavity, GTV, and PTV1. The dose was normalized to the prescribed dose of GTV and the threshold criteria used to evaluate the difference in planar dose were 3% and 3 mm.

Setup of all measurement was accurately aligned by an alignment laser. IMRT and VMAT measurements were accompanied by alignment using the six degree-of-freedom image-guided cone-beam CT to confirm the position. MVCT was utilized to align the tomotherapy measurements and correct the position shifts and roll-angle deviations in the rotating gantry.

## 3. Results

### 3.1. Calculation of Results of Treatment Plans

The *V*_95%_ of GTV obtained for IMRT, VMAT, and tomotherapy were all 100%. The *V*_95%_ of PTV1 obtained for IMRT, VMAT, and tomotherapy were 96%, 96%, and 100%, respectively, and the *V*_95%_ of PTV2 were 95%, 97%, and 99.9%, respectively. *D*_max_ of the brainstem were 3899 cGy, 3806 cGy, and 3574 cGy, respectively, while the *D*_max_ of the spinal cord were 3597 cGy, 3228 cGy, and 3257 cGy, respectively. *D*_mean_ of the right parotid gland were 2209 cGy, 2174 cGy, and 2387 cGy, respectively, and *D*_mean_ of the left parotid gland were 2101 cGy, 2057 cGy, and 2064 cGy, respectively (Table 1). All plans complied with the standards of clinical treatment [17].

### 3.2. Dose Measurements of Dosimeters

#### 3.2.1. Dose Measurements of Single Field and Parallel Opposed Fields

**Figure 3:**
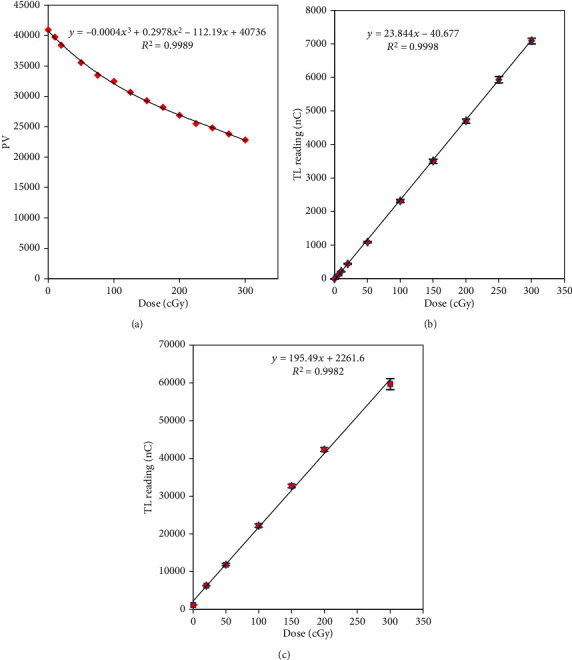
Dose-linearity curves for 6 MV: (a) EBT3, (b) TLD-100, and (c) GR-200F.

**Figure 4:**
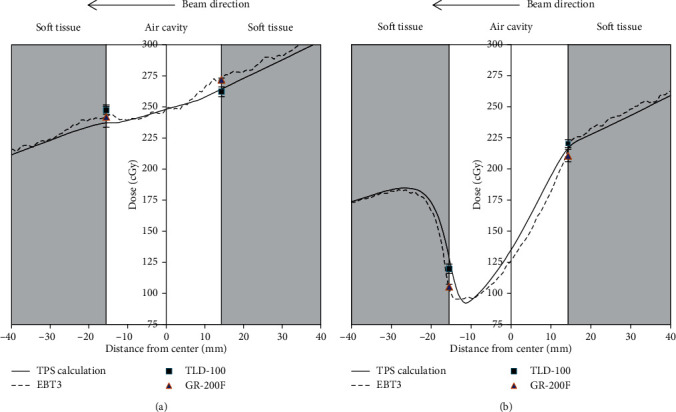
Central axis dose distribution of the nasopharyngeal air cavity under single field radiation (a) 10 × 10 cm^2^ and (b) 2 × 2 cm^2^.

Figure 3 shows the dose-linearity curves of EBT3, TLD-100, and GR-200F for 6 MV photon beams (0–300 cGy); the *R*-square values of all dose-linearity curves were above 0.99. The central axis depth dose distribution in the nasopharyngeal cavity under single field radiation is shown in Figure 4. The path of the beam through the nasopharyngeal cavity was 3 cm. The cavity width along the superior-inferior direction at the cavity center was 4 cm, and the height along the anterior-posterior direction was 5.75 cm.

When the irradiation was a 10 × 10 cm^2^ single field beam, the differences between the average dose of the measurement beam at the proximal interface for EBT3, GR-200F, and TLD-100 and the calculated values of Pinnacle TPS were 3.5%, 2.9%, and−0.8%, respectively. The differences between the average dose of the measurement beam at the distal interface and the TPS compared to the calculated values were 1.9%, 2.3%, and 3.3%, respectively. Thus, the measurement values for EBT3, GR-200F, and TLD-100 approached those of the TPS calculation values, and the differences were all within 3.5%. Furthermore, the distal interface was not subjected to a rebuild-up caused by the electronic disequilibrium effect. This indicates that the electronic disequilibrium effect was not significant under a large field, and that the TPS could accurately estimate the dose at the air-tissue interface under a large field.

When the irradiation was a 2 × 2 cm^2^ single field beam, the differences between the average dose measured at the proximal interface for EBT3, GR-200F, and TLD-100 and the calculated values of Pinnacle TPS were −1.2%, −3.5%, and 0.8%, respectively. The differences between the average dose measured at the distal interface and the calculated dose of Pinnacle TPS were−15.8%, −16.4%, and−4.9%, respectively. The EBT3 and GR-200F measurements were similar, and both results should closely reflect the actual interface dose due to their ultrathin thickness. However, these EBT3 and GR-200F results indicate that CCCS algorithms severely underestimate the dose at the air-tissue interface. Due to the dosimeter thickness of the TLD-100, the measurement values of the average dose that are within its thickness levels (0.38 mm) would not reflect the actual interface dose.

**Figure 5:**
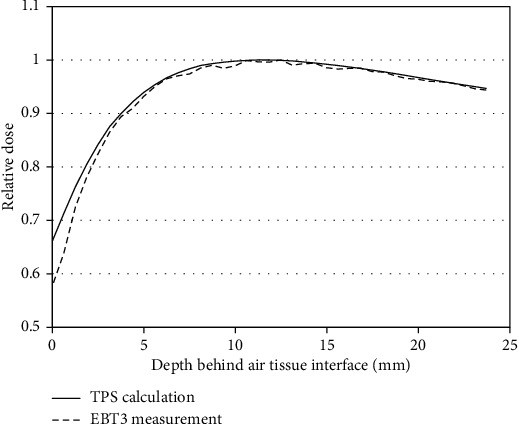
Relative dose of the depth behind the air-tissue distal interface under irradiation of the nasopharyngeal cavity using a 2 × 2 cm^2^ single field beam.

Figure 5 shows the relative dose of the depth behind the air-tissue interface of 2 × 2 cm^2^ single field. The BUR^−1^ of the air-tissue interface, calculated using Pinnacle TPS, was 0.66, and the BUR^−1^ measured using EBT3 was 0.58. Hence, the calculated TPS value overestimated the value measured by 13.8%.

**Figure 6:**
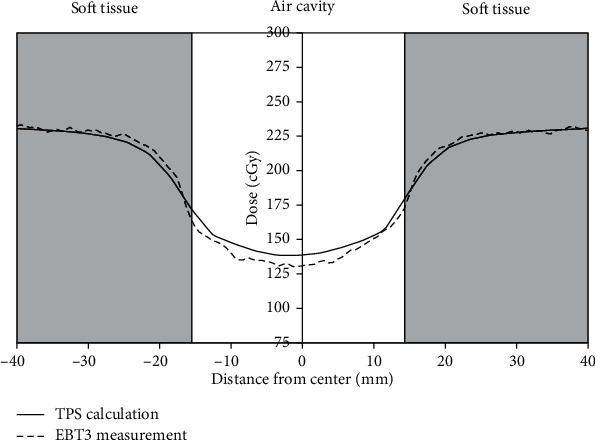
Dose distribution under irradiation of the nasopharyngeal air cavity by two bilateral opposed 2 × 2 cm^2^ fields.

Figure 6 shows two 2 × 2 cm^2^ fields, which are opposing parallel irradiation fields at beam directions of 90° and 270°, respectively. The EBT3 results indicate that the difference between the measured values and the calculated values was reduced to −3.7% and−4.2%, respectively, for the left and right air-tissue interfaces. This implies that beam dispersion can reduce the impact of the electronic disequilibrium effect, thus decreasing the magnitude of TPS dose overestimation.

#### 3.2.2. Dose Measurements of Multiple Fields in Clinical Treatment Plans

Table 2 shows the results measured using TLD-100 and GR-200F at five dose points on the tissue interface, the difference between these measurements, and the calculated TPS values. The quadratic means (QM) of the percentage errors determined using TLD-100 and GR-200F values for the IMRT treatment plan were 2.3% and 2.1%, respectively; those for the VMAT treatment plan were 2.3% and 4.0%, respectively, while those for the tomotherapy treatment plan were both 3.1%. For IMRT, the GRF-200F measurements indicated that the TPS at point 5 and tomotherapy at point 1 were overestimated by 4.2% and 4.3%, respectively, while the remaining points were not significantly underdosed. Overall, TLD-100 and GR-200F did not show significant differences in the measurement of treatment plans using three clinical treatment techniques. This implies that these clinical treatment techniques did not form significant rebuild-up regions at the nasopharyngeal air-tissue interface. Moreover, the measured results of TLD with different thickness under clinical applications of multi-angle irradiation did not significantly different from the TPS calculations.

**Figure 7:**
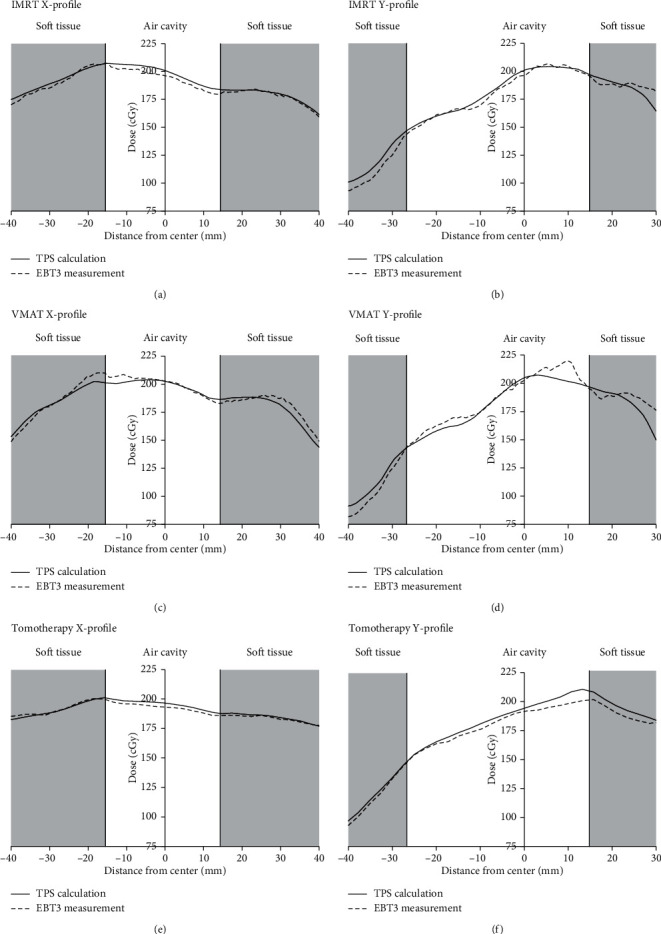
Profiles of different techniques measured using EBT3 at the nasopharyngeal air cavity on the coronal plane. X-profile represents the profile in the left-right directions of the body. Y-profile represents the profile in the superior-inferior directions of the body. (a) X-profile of IMRT; (b) Y-profile of IMRT; (c) X-profile of VMAT; (d) Y-profile of VMAT; (e) X-profile of tomotherapy; and (f) Y-profile of tomotherapy.

The dose profiles of the phantom cavity on the coronal plane, measured using EBT3 with the beam direction along the left-right directions of the body (*x*-axis), are presented in Figure 7. The maximum difference measured at the interface along the *x*-axis was for VMAT, where the differences in the right and left interface doses between the measured and calculated values were 4.2% and −1.92%, respectively. The maximum difference measured at the interface along the superior-inferior directions of the body (*y*-axis) was for tomotherapy, where the differences between the measured and calculated values of the right and left interface doses were −2.52% and −3.6%, respectively. Overall, the results show that the treatment plans for these three techniques did not significantly overestimate the dose at the air-tissue interface.

**Figure 8:**
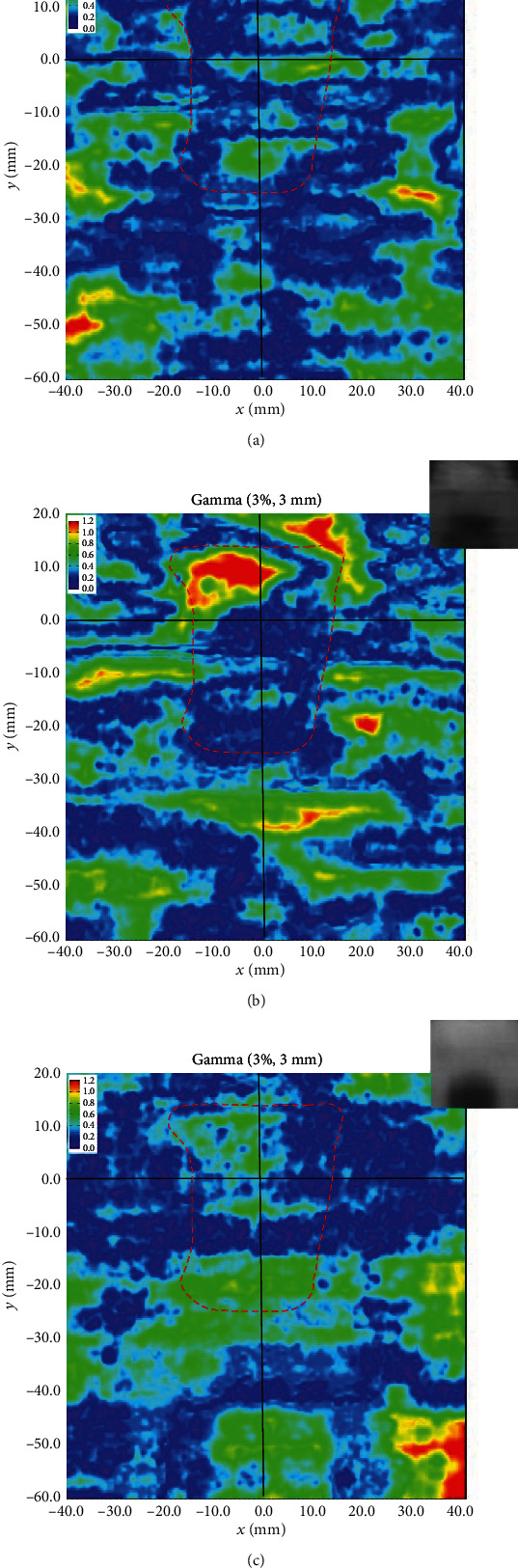
Gamma evaluation maps obtained using the cavity doses on the coronal plane measured by EBT3 within the phantom. (a) IMRT, (b) VMAT, and (c) tomotherapy. (The red dashed curve indicates the shape of the nasopharyngeal cavity).

Figure 8 shows the gamma evaluation maps for nasopharyngeal cavity on the coronal plane, measured within the phantom by EBT3; the gamma criteria were 3% and 3 mm. The gamma pass rates measured for IMRT, VMAT, and tomotherapy were 98.5%, 94.1%, and 97.3%, respectively. These results indicate that, despite the presence of a cavity, EBT3 measurements still showed high pass rates. However, certain fail regions were observed in the nasopharyngeal cavity for VMAT, as indicated by higher measurement dose compared to the calculated values. The higher measurement dose might be due to the error of MLC position rather than electronic disequilibrium effect. Previous study had revealed that it will cause significant impact on the dose even the error of MLC position less than 1 mm [18, 19]. The results measured for all three clinical techniques did not show evidence of fail regions that would be caused by significant dose overestimation at the nasopharyngeal air-tissue interface.

## 4. Discussion

The differences in the measured and calculated values at the proximal interface were smaller than 3.5% with regard to the 2 × 2 cm^2^ small single field. This is because the main contributors to the dose at the proximal interface are the primary beam and phantom scatter. The presence of the nasopharyngeal cavity would reduce the contribution to proximal interface by backscatter dose because of the low density of the air cavity. However, compared to the distal interface, the dose perturbation at the proximal interface caused by the electronic disequilibrium is less than that at the distal interface due to its sufficient forward and lateral scattering. With regard to the distal interface, however, there was significant electronic disequilibrium that led to the formation of rebuild-up regions, with differences between the measured and calculated values at the interface of up to 16.4%.

Kan et al. used TLD to measure the BUR^−1^ of a phantom containing a 3 × 3 × 30 cm^3^ cavity under a 6 MV beam with a 2 × 2 cm^2^ field; the BUR^−1^ measured in that study was 0.34 [20]. Martens et al. used the MD 55 Gafchromic film to measure the BUR^−1^ of a phantom containing a cylindrical cavity with a 2 cm diameter under a 6 MV beam with a 10 × 2 cm^2^ field [21]. The BUR^−1^ measured in that study was 0.74, and Pinnacle TPS overestimated the dose at this cavity by 9%. BUR^−1^ may differ due to differences in beam energy, cavity volume, and field size. As our study results are based on simulations of the shape and size of an actual human nasopharyngeal cavity, the cavity size was smaller than that used in the studies mentioned above. Hence, our results are more closely aligned to the actual effect caused by the nasopharyngeal cavity.

The primary reason for the dose overestimation of TPS calculation values at the air-tissue interface is that the CCCS algorithms used in the TPS perform distribution expansion of the cavity kernel based on density scaling. However, the magnitude of expansion may be underestimated, which leads to an underestimation of the lateral electronic disequilibrium effect at the air-tissue interface. This will in turn cause the TPS to overestimate the dose deposit at the interface under a small field. Due to the differences in dose contributions by lateral electronic scattering, the electronic disequilibrium effect at an interface with perpendicular beam incidence will be more severe than an interface parallel to the beam. Kan et al. showed that the rebuild-up effects at the interface due to electronic disequilibrium caused by the lack of lateral electronic scatter are negligible when the field size is greater than 4 × 4 cm^2^ [22].

Wang et al. employed the Monte Carlo method to calculate the treatment plan doses of head-and-neck cancer patients receiving 3D conformal radiotherapy [23]. These calculated doses were then compared to the original treatment plan calculation based on the pencil beam algorithm. Their results showed that there were no significant differences between the two algorithms in clinical 3D conformal radiotherapy. Our results also showed that the interface doses for IMRT, VMAT, and tomotherapy were not overestimated due to electronic disequilibrium, and the measured differences in dose were all within the clinical uncertainty range of dose measurements (±5% recommended by AAPM TG-119 and TG-142) [24, 25]. This was primarily because the beam angles specified in the clinical treatment plans were multiangle radiation beams. In terms of the air-tissue interface, radiation doses that passed through the cavity and were incident perpendicularly on the tissue interface, with field sizes smaller than 4 × 4 cm^2^, only accounted for a small weighting of the total prescribed dose. Hence, the resultant magnitude of the reduction in dose was minute, and the resulting dose errors were still within 5%.

Chen et al. showed that the outcomes of IMRT and 3DCRT were similar considering locoregional control (LRC) for early-stage patients [26]. IMRT was associated with better LRC compared with 3DCRT for advanced-stage patients. The result of clinical follow-up also revealed that the nasopharyngeal cavity has no significant effect on the outcome of the IMRT treatment.

## 5. Conclusions

This study examined the accuracy of CCCS algorithms in calculating the interface dose under a simple small field in a simulated model that closely aligned to the actual human nasopharyngeal profile. In addition, this study also measured the accuracy of the algorithm at the nasopharyngeal cavity and soft tissue interface, when applied to IMRT, VMAT, and tomotherapy in the clinical treatment of early stage NPC that the air cavity would not be filled up by tumor and the maximum cavity volume could be maintained. Under single small fields and parallel opposed fields, the measurement results indicated that the TPS overestimated the interface dose. However, measurements of IMRT, VMAT, and tomotherapy revealed that the measurement values at the air-tissue interface were not significantly lower than the calculated values. This indicates that under clinical multiangle irradiation, the dose errors from these calculations, due to the small-field electronic disequilibrium effect, are negligible when compared to the total prescribed dose. Nevertheless, to further reduce the impact of electronic disequilibrium caused by small-field size at the air-tissue interface, the following measures can be taken: (a) limit the minimum area of subfields when formulating treatment plans, (b) prescribe the dose using multiangle beams whenever possible, (c) reduce the dose weighting of beams entering the concerned interface through the cavity, and (d) use beam eye view to examine the area of each subfield; if the subfield area of beams entering the tissue interface through the cavity is too small or unilaterally too narrow, then these can be removed manually or the subfield area can be enlarged.

## Figures and Tables

**Table 1:** Dose summary of target and organ at risk calculated by IMRT, VMAT, and tomotherapy.

	IMRT	VMAT	Tomotherapy
*V*_95%_ (GTV)/%	100	100	100
*V*_95%_ (PTV1)/%	96.0	96.0	100
*V*_95%_ (PTV2)/%	95.0	97.0	99.9
*D*_max_ (brainstem)/cGy	3899	3806	3574
*D*_max_ (spinal cord)/cGy	3597	3228	3257
*D*_mean_ (R't parotid gland)/cGy	2209	2174	2387
*D*_mean_ (L't parotid gland)/cGy	2101	2057	2064

**Table 2:** Dosage measured by TLD-100 and GR-200F for IMRT, VMAT, and tomotherapy at the air-tissue interface.

	Position	TPS calculation (cGy)	TLD-100 measurement (cGy)	GR-200F measurement (cGy)	TLD-100 difference (%)	GR-200 difference (%)
IMRT	1	167.8	161.2 ± 2.7	168.9 ± 1.6	−3.9	0.7
	2	208.0	202.2 ± 2.6	207.1 ± 0.3	−2.8	0.4
	3	184.6	188.0 ± 7.9	187.4 ± 4.8	1.8	1.5
	4	167.1	165.4 ± 2.7	169.0 ± 0.5	−1.0	1.1
	5	198.7	199.1 ± 4.7	190.3 ± 1.3	−0.5	−4.2
	QM				2.3	2.1

VMAT	1	169.1	173.3 ± 2.6	176.6 ± 4.7	2.5	4.4
	2	207.3	214.0 ± 5.4	219.4 ± 7.4	3.2	5.8
	3	190.0	192.3 ± 5.2	197.8 ± 5.4	1.2	4.1
	4	169.2	168.4 ± 2.7	170.3 ± 1.8	−0.5	0.7
	5	202.2	208.0 ± 9.1	208.5 ± 5.7	2.9	3.1
	QM				2.3	4.0

Tomotherapy	1	171.6	165.9 ± 2.0	164.3 ± 8.6	−3.3	−4.3
	2	204.1	201.2 ± 3.9	209.8 ± 13.6	−1.4	2.8
	3	188.4	184.1 ± 3.6	190.2 ± 7.3	−2.3	1.0
	4	156.03	150.1 ± 2.7	156.9 ± 7.3	−3.8	0.6
	5	185.6	178.3 ± 1.9	194.1 ± 6.3	−3.9	4.6
	QM				3.1	3.1

## Data Availability

The data used to support the findings of this study are available from the corresponding authors upon request.
